# Desiccation Tolerance in Chlorophyllous Fern Spores: Are Ecophysiological Features Related to Environmental Conditions?

**DOI:** 10.3389/fpls.2019.01130

**Published:** 2019-09-20

**Authors:** Marina López-Pozo, Daniel Ballesteros, José Manuel Laza, José Ignacio García-Plazaola, Beatriz Fernández-Marín

**Affiliations:** ^1^Depatment of Plant Biology and Ecology, University of the Basque Country (UPV/EHU), Bilbao, Spain; ^2^Comparative Plant and Fungal Biology, Royal Botanic Gardens, Kew, West Sussex, United Kingdom; ^3^Laboratory of Macromolecular Chemistry (Labquimac), Department of Physical Chemistry, University of the Basque Country (UPV/EHU), Bilbao, Spain

**Keywords:** desiccation tolerance, dynamic mechanical analysis, environmental conditions, glassy state, green spores, molecular mobility, tocopherol, water relations

## Abstract

Fern spores of most species are desiccation tolerant (DT) and, in some cases, are photosynthetic at maturation, the so-called chlorophyllous spores (CS). The lifespan of CS in the dry state is very variable among species. The physiological, biochemical, and biophysical mechanisms underpinning this variability remain understudied and their interpretation from an ecophysiological approach virtually unexplored. In this study, we aimed at fulfilling this gap by assessing photochemical, hydric, and biophysical properties of CS from three temperate species with contrasting biological strategies and longevity in the dry state: *Equisetum telmateia* (spore maturation and release in spring, ultrashort lifespan), *Osmunda regalis* (spore maturation and release in summer, medium lifespan), *Matteuccia struthiopteris* (spore maturation and release in winter, medium-long lifespan). After subjection of CS to controlled drying treatments, results showed that the three species displayed different extents of DT. CS of *E. telmateia* rapidly lost viability after desiccation, while the other two withstood several dehydration–rehydration cycles without compromising viability. The extent of DT was in concordance with water availability in the sporulation season of each species. CS of *O. regalis* and *M. struthiopteris* carried out the characteristic quenching of chlorophyll fluorescence, widely displayed by other DT cryptogams during drying, and had higher tocopherol and proline contents. The turgor loss point of CS is also related to the extent of DT and to the sporulation season: lowest values were found in CS of *M. struthiopteris* and *O. regalis*. The hydrophobicity of spores in these two species was higher and probably related to the prevention of water absorption under unfavorable conditions. Molecular mobility, estimated by dynamic mechanical thermal analysis, confirmed an unstable glassy state in the spores of *E. telmateia*, directly related to the low DT, while the DT species entered in a stable glassy state when dried. Overall, our data revealed a DT syndrome related to the season of sporulation that was characterized by higher photoprotective potential, specific hydric properties, and lower molecular mobility in the dry state. Being unicellular haploid structures, CS represent not only a challenge for germplasm preservation (e.g., as these spores are prone to photooxidation) but also an excellent opportunity for studying mechanisms of DT in photosynthetic cells.

## Introduction

The terrestrial atmosphere is, in general, an ambient desiccant, and therefore, the organisms that inhabit here run the risk of drying out if they do not control water loss (Oliver and Bewley, 1997). To face this challenge, some plants have developed a survival strategy, known as “desiccation tolerance” (DT). This allows the recovery of full metabolism when the organisms are rehydrated after being desiccated to low water contents (WC), usually those below 0.1 g H_2_O g^−1^ DW or those in equilibrium at relative humidity (RH) <50% (Proctor and Tuba, 2002, Leprince and Buitink, 2010; Fernández-Marín et al., 2016). 

DT is frequent in reproductive structures (seeds, pollen, and spores) (Gaff and Oliver, 2013), but its occurrence among vegetative photosynthetic tissues is much more limited (Fernández-Marín et al., 2016). There is increasing recognition in variation in the responses of diverse plant reproductive structures to desiccation (e.g., Berjak and Pammenter, 2008; Franchi et al., 2011; Walters, 2015; López-Pozo et al., 2019). This variation has been mainly studied in seeds, where distinction between “fully” DT and desiccation sensitive (DS) seeds (commonly expressed in the duality orthodox versus recalcitrant seeds) is complemented with the presence of seeds showing an intermediate tolerance to drying (Walters, 2015). In the case of other reproductive structures, such as spores, the same intermediate tolerance may be found. Ferns produce two different types of spores: chlorophyllous spores (CS) and non-CS. The former have fully developed photosynthetic machinery once mature, being able to start photosynthesis immediately once hydrated (Sundue et al., 2011; López-Pozo et al., 2019). The presence of chloroplasts, and hence of chlorophyll, makes CS particularly sensitive to photooxidative damage (Ballesteros et al., 2018). During desiccation and in the dry state, CS must prevent light–chlorophyll interaction avoiding the production of reactive oxygen species (ROS) (Kranner et al., 2002; Ballesteros et al., 2018). 

Fern spores are generally DT, but their lifespan in the dry state may vary among species, probably due to different extents of DT (Ballesteros et al., 2017). Notwithstanding the above, most of them are non-CS (López-Pozo et al., 2018). Spores are the main dispersal structure in ferns. Upon germination, spores give rise to the gametophytic generation where sexual reproduction occurs. Therefore, the ecological success of the pteridophytes will depend on the ability of their spores to disperse and survive under various environmental conditions, with controlling water loss as one of the most critical factors (Page, 2002). To date, there are no studies on how fern spores survive natural environmental fluctuations (particularly moisture fluctuations), and less is known about the importance that some features can have in the temporal or spatial distribution of species.

The mechanisms that confer DT and longevity in fern CS (or other chlorophyllous reproductive structures such as chlorophyllous seeds) have been rarely studied, and large gaps of knowledge still exist (Roqueiro et al., 2010; Ballesteros et al., 2018). For example, to our knowledge, only one study has investigated some molecular mechanisms of DT in CS. This study discovered the expression of late embryogenesis abundant (LEA) proteins during the maturation stage of the CS of *Onoclea sensibilis* in the sporangia and highlighted the role that these LEA proteins may have in the acquisition of DT and the survival of the desiccation stage (Raghavan and Kamalay, 1992). Most of the studies available have focused on the interaction of temperature and moisture content once spores are dried and how they control longevity of the CS, often for *ex situ* conservation purposes (Pence, 2000; Ballesteros and Walters, 2011; Magrini and Scoppola, 2012; Li and Shi, 2014; Li and Shi, 2015; Mikula et al., 2015; Ballesteros et al., 2017, Ballesteros et al., 2018, Ballesteros et al., 2019). However, the degree of DT in CS can be inferred from them, since, in the great majority of studies, the spores are dehydrated for later storage. Even so, some experiments focused on the direct response to dehydration, freezing, or light (Kato, 1976; Lebkuecher, 1997; Ballesteros et al., 2018; López-Pozo et al., 2019). All studies showed to some extent that the damage to spores was exacerbated according to the severity of desiccation and that deterioration was greater in CS than in non-CS. This variation suggests that the structural and physicochemical stability of the dry state differs according to the species and the type of spore, e.g., CS or non-CS (e.g., Ballesteros et al., 2017, Ballesteros et al., 2019). The unicellular structure of CS makes them a good unicellular model to investigate DT and longevity in the dry state. The knowledge generated can be used to understand what happens in more complex systems, such as seeds and photosynthetic vegetative tissues, in which the presence of multiple cells and tissues can confound the interpretation of the results obtained (Ballesteros et al., 2017; López-Pozo et al., 2018; Ballesteros et al., 2019; López-Pozo et al., 2019). In addition, the intermediate position of ferns in the plant phylogeny (between the bryophytes and the seeded plants) can be useful to understand the evolution of DT across different plant lineages (López-Pozo et al., 2018). 

Available literature on DT (non-chlorophyllous) seeds and in leaves of resurrection plants has already revealed some common mechanism among DT tissues. When desiccation is completed, cell content is stabilized by solidification, and it enters into a glassy state (Walters et al., 2010). At these conditions, differences in the properties of the solid formed (i.e., differences in the glassy state), and the short-range molecular mobility allowed within the solid matrix (including enzymatic activity) will regulate life in the dry state and will be responsible for longevity (Buitink et al., 1998, Buitink et al., 2000; Walters et al., 2010; Ballesteros and Walters, 2011; Ballesteros and Walters, 2019; Ballesteros et al., 2019). Two major divisions can be observed in DT organisms; those who dismantle the photosynthetic apparatus upon desiccation, known as poikilochlorophyllyllous, and those who maintain its integrity, known as homoiochlorophyllyllous (reviewed in Fernández-Marín et al., 2016). The first is rarely found among DT plants, requires more time to reactivate metabolism during rewatering, and is restricted to vascular plants. Basically, poikilochlorophyllyllous plants degrade chlorophyll and the chloroplast ultrastructure when water availability starts to be limited. Homoiochlorophyllyllous DT plants, instead, survive desiccation without this machinery degradation and have to cope with the oxidative damage occurred during the absence of water (Fernández-Marín et al., 2016). Based on these characteristics, it has been suggested that CS spores may act as homoiochlorophyllyllous organisms and non-CS spores as poikilochlorophyllyllous (Ballesteros et al., 2018). The mechanisms that allow DT in homoiochlorophyllyllous organisms preserve the cellular integrity during drying and in the dry state by avoiding the disruption of ultrastructures and by counteracting the oxidative damage that results from the generation of ROS and free radicals (Dinakar et al., 2012; Walters, 2015). Among these mechanisms, some of the most universal responses are the accumulation of compatible osmolytes (Bartels and Sunkar, 2005), the accumulation of specific compounds such as LEA proteins or heat shock proteins, and an efficient thermal energy dissipation (in the case of dry chlorophyllous cells). The osmotic adjustment and changes in membrane lipids composition are also essential mechanisms to cope with water loss (Gasulla et al., 2013; Fernández-Marín et al., 2016). When desiccation begins, a decrease in chlorophyll fluorescence emitted by photosystem II (PSII) has been reported in several DT organisms with chlorophyllous cells (Lebkuecher, 1997; Heber et al., 2007; López-Pozo et al., 2019). This mechanism, the so-called nonphotochemical quenching induced by desiccation (NPQd), plays a central photoprotective role by reemitting heat as excess light energy absorbed by chlorophyll (Bilger, 2014). It is often correlated with the synthesis of zeaxanthin (Fernández-Marín et al., 2011) but can also be induced in the absence of activation of the xanthophyll (VAZ) cycle (Heber et al., 2010). Regarding mechanical stress, osmotic adjustment is essential for the proper functioning of the cell (Dinakar et al., 2016). Maintaining turgor prevents mechanical and biochemical damage at the cellular level, preserving cellular integrity (Fernández-Marín et al., 2016). An important molecule in cellular osmotic adjustment is proline, which has a double function: preservation of cell turgor (Bartlett et al., 2014; Fernández-Marín et al., 2016) and removal of ROS (Borsani et al., 1999). 

Our aim in this multidisciplinary work is to study the biochemical and physiological features that may be involved in the variation of responses to desiccation of CS of three different fern species, as well as in relation to their ecophysiological significance. The three CS studied (*Matteuccia struthiopteris*, *Osmunda regalis*, and* Equisetum telmateia*) differ in the extent of DT and longevity in the dry state as well as in the ecological niche that they occupy (Ballesteros et al., 2017). We hypothesize that CS with higher DT will show more stable physicochemical features. In addition, we hypothesize that those that are more DT will be released in the more unfavorable environmental conditions, whereas those less DT will mature in more favorable environmental conditions.

## Materials and Methods

### Plant Material

CS of the three fern species studied were obtained from mature fertile fronds. *M. struthiopteris* (*MSt*) CS were obtained from outdoor individuals from the Botanical Garden of the University of Innsbruck, Austria (47°16′ N, 11°23′ E, 600 m a.s.l.) in December 2016. *E. telmateia* (*ETe*) horsetails growing along Gobelas river, Getxo, Spain (43°20′32.31″ N, 3°0′0.20″ W) were sampled in March 2017. *O. regalis* (*ORe*) CS were obtained from the surroundings of the University of the Basque Country, Leioa, Spain (43°19′48.8″ N 2°58′08.5″ W) in June 2017.

After collection, *ORe*,* MSt*, and *ETe* fronds were kept in the laboratory at 60% relative humidity (RH) for 24 h allowing the dehiscence of the sporangia and the release of the spores. Once the spores were released, they were separated in aliquots of 50 mg, immersed in liquid N_2_, and stored at −80°C until the beginning of the experiment. Before use, spores were defrosted in a water bath at 35°C for 5 min (Pence, 2008).

### Determination of Photosynthetic Pigments and Tocopherols

Approximately 15 mg of frozen spores per replicate (exact weight recorded) were freeze dried and then used for the characterization of photosynthetic pigments and tocopherols in the sporangial state. Samples were first extracted in acetone/water (95:5) and second in pure acetone, both buffered with CaCO_3_. Freeze-dried samples were homogenized with a mill, and the extracts were centrifuged at 16,100 *g* for 20 min. Finally, supernatants were filtered through a 0.2-µm polytetrafluoroethylene filter (Teknokroma, Barcelona, Spain) before being analyzed by high-performance liquid chromatography (HPLC). Extracts were injected (15 µl) into a reverse-phase Waters (Milford, MA, USA) HPLC system following the method of García-Plazaola and Becerril (1999) with the modifications described in García-Plazaola and Becerril (2001). Photodiode array detector (Waters model 996) was used to measure photosynthetic pigments in the range of 250–700 nm, and peaks were detected and integrated at 445 nm for chlorophyll content. Pigments were identified and quantified by the method described by García-Plazaola and Becerril (1999). Retention times and conversion factors for pigments were the same as described by García-Plazaola and Becerril (1999) and García-Plazaola and Becerril (2001). 

### Proline Determination

Proline content was extracted from ∼10 mg of spores in their water (MCW) (12:5:1) according to Borsani et al., (1999). The homogenized was centrifuged for 2 min at 2,000 *g* and another milliliter of MCW added. After vortexing vigorously, 1 ml chloroform and 1.5 ml water were added. Two phases were generated where the upper phase corresponds to free proline. This phase was mixed with ninhydrin reagent, warmed at 100°C for 1 h and left at room temperature to cool down. Finally, 2 ml of toluene was added, vortexed, and centrifuged at maximum speed during 10 min. Proline determination was measured by spectrophotometry at 515 nm (Troll and Lindsley, 1955).

### Extent of Desiccation Tolerance in CS

To estimate the extent of DT, two consecutive desiccation–rehydration cycles (D–R) were conducted in aliquots of 50 mg spores under 10% RH and under 100% RH. Treatments were performed in hermetically closed chambers of 5 L volume; 10% RH was achieved placing 400 g of silica gel (Labkem, SGE0-002-3K0) in the bottom of the hermetically closed chambers. The large volume of the chambers compared to the amount of spores avoided O_2_ limitation. Spores released from the sporangium and hydrated during 24 h in darkness were considered as “controls.” Spores from mature fronds of each fern species (not subjected to any previous hydration) were placed in Eppendorf tubes of 2 ml and kept under 10% RH during 36 h in darkness. After this first dehydration (D1), distilled water was added to the spores, and they were incubated under 100% RH for 24 h, considering this as the first rehydration (R1). Once this period of 24 h was finished, second dehydration (D2) was performed at 10% RH in darkness. Owing to the higher amount of water in spores than in the first desiccation, 72 h were needed for the complete dehydration. Finally, the second hydration (R2) was carried out with the same procedure as the first one. 

All the treatments for all species were performed in darkness. Chlorophyll fluorescence was measured after both desiccations (D1 and D2) and rehydrations (R1 and R2) and water content (WC) after both D1 and D2.

#### Chlorophyll Fluorescence

Chlorophyll fluorescence in spores was measured using a pulse amplitude-modulated fluorimeter (PAM 2500, Walz, Effeltrich, Germany). The optical fiber was fixed off to maintain the same distance to the samples in all measurements. The minimum chlorophyll fluorescence (Fo) was determined in dark-adapted (≤30 min) spores. The maximum chlorophyll fluorescence (Fm) was induced with a saturating pulse for 500 ms. The variable chlorophyll fluorescence (Fv) was calculated as Fm–Fo. The ratio Fv/Fm represents the maximum photochemical efficiency of PSII. 

#### Water Content

To determine the amount of water in spores after being released from the frond (sporangial state) and after each desiccation (D1 and D2), the WC of samples was estimated as follows:

$$WC=\frac{\left(EW-DW\right)}{DW}$$

where EW is the weight at the equilibrium with RH, and DW is the weight after drying for 24 h in an oven at 70°C. Samples were weighed using a balance (AB104, Mettler Toledo, Barcelona, Spain) with 0.1 mg accuracy.

#### Germination Percentage

To determine the viability of spores after the D–R cycles, spores were sown on Dyer culture medium (Dyer, 1979). Percentage of germination was examined 10 days after sowing. The estimation of germination rate was carried out by counting random samples of ∼100 spores per Petri dish. Mature spores without treatment were sown on Dyer medium during 10 days at 20 ± 2°C, with 12 h/12 h of photoperiod and a photosynthetic photon flux density of 70 μmol m^−2^ s^−1^ were germination control values. Germination was considered complete once the spore coat had broken and the rhizoids had emerged (Ballesteros et al., 2012).

#### Molecular Mobility

Mechanical properties of spores equilibrated at 10% RH for 48 h were measured using a dynamic mechanical thermal analyzer (DMA/SDTA861e, Mettler Toledo, Greifensee, Switzerland DMTA). Shear tests required the production of circular samples ≤2 mm thick and 13 mm in diameter. For that purpose, 300 mg (150 mg per circular sample) of spores dehydrated at 10% RH were compressed in a hydraulic press using a maximum pressure of 10 t. All tests were carried out in the dynamic mode, from −50 to 150°C at a heating rate of 2°C min^−1^ (Fernández-Marín et al., 2013). The analysis of molecular mobility provides information about the potentiality of enzymatic activity at a wide range of temperature. Shear storage modulus (G´) and the loss tangent (tan δ) were calculated using the Mettler Toledo STARTe software during dynamic mechanical thermal analysis (DMTA) scans. For each biological replicate, the temperature value at the maximum tan δ coincident with the α-relaxation measured at 1 Hz was considered for glass transition temperature (Tg) estimation. The Tg indicates that the spore cytoplasm changes from a solid state (known as “glassy state”) to a more fluid state upon warming, which allows diffusive motion (Buitink et al., 1996; Ballesteros and Walters, 2011). The extent to which relaxations released restricted motion was approximated by the size of the relaxation signal and was calculated from the difference in value of the tan δ at the onset and peak of α-relaxation (Ballesteros and Walters, 2011).

### Water Relations

#### Pressure–Volume Curves

Water potentials of CS were measured using a dewpoint hygrometer (WP4, Decagon Devices, Pullmann, WA, USA). Around 200 mg of spores was placed in the psychrometer cuvette and held at 100% RH for 48 h in darkness (for germination avoidance) in closed chambers to allow rehydration. After this period, the samples were considered fully hydrated and ready for water potentials measurements. Previous to the first measurement, the instrument was calibrated with a standard solution of KCl (0.5 M).The first measurement corresponded to the initial water potential. Once the first reading was complete, the sample was weighed on a balance, corresponding to total fresh weight. Consecutive measurements were made on each replicate, weighing them after each reading. Between readings, the samples were kept in the lab at 50% RH, allowing the loss of water. Samples for last readings were kept over silica gel between measurements to force the loss of water. After the measurements, samples were then oven dried at 70°C for 24 h and their DW determined.

The results were analyzed by two graphs: the linear plot of water content against water potential and the pressure–volume (P–V) curve relating the reciprocal of the water potential to water content. In the first measurements, a high amount of water loss could be observed but not a decline in water potential (Ψ). As the samples were losing water, the water potentials began to fall, at first slowly without much change, until reaching a point of abrupt change, considered the water potential at turgor loss point (Ψ_TLP_). The experiment was concluded when the relationship between 1/Ψ and the cumulative amount of water loss by samples became linear (r^2^ > 0.98). From P–V curves, several parameters were obtained, such as relative water content at turgor loss point (RWC_TLP_), saturated water content (SWC), water potential at full turgor (Ψ_O_), water potential at turgor loss point (Ψ_TLP_), water potential in the sporangial state (Ψ_SPO_), and the modulus of elasticity, known as epsilon (ε) (Sack et al., 2011). Water potential in the sporangial state was inferred through the adjustment of the trend line in the regression curve between Ψ and WC. The curve of each replicate was adjusted to a polynomial of degree 4. Sporangial WC values were substituted in the equation.

#### Analysis of Rehydration Kinetics *via* the Coat Surface 

Static contact angle (CA) and its change during time were used for the interpretation of the rehydration capacity of CS in their sporangial state (e.g., mature spores at the stage in which they are naturally released from sporangia). For each replicate, a monolayer of spores was mounted over a microscope slide and fixed with a double-sided cellophane. An optical CA measuring instrument (OCA 15EC, from Data Physics Instruments GmbH, Filderstadt, Germany) was used to define the hydrophobicity of the spore surface. A sessile droplet of 6 µl distilled water was released over the spores of *ORe*, *ETe*, and *MSt* and changes in its CA recorded at a speed of 30 frames s^−1^, during 10 s, with a charge-coupled device equipped camera. CAs were measured with the SCA software for OCA, v.4.4.3., as the mean from the optically left and right margins of the water droplet in each frame. The measuring precision was of ±0.1°. Measurements were performed in 10 different replicates per species.

### Scanning Electron Microscopy

Spores in their sporangial state without any desiccation treatment were used for scanning electron microscopy (SEM) analysis, placing them on adhesive disks pasted on stubs (Agar Scientific Ltd, Essex, UK), and gold coated for 10 min with a fine coat ion sputter (JFC-1100, Tokyo, Japan). Spores were observed using a scanning electron microscope (Hitachi S-4800, Hitachi Ltd., Tokyo, Japan) with an average working distance of 8 mm and a voltage of 10 kV. 

### Statistical Analysis

Kolmogorov–Smirnov and Levene tests were used respectively to check for normal distribution and homogeneity of variance of the data. One-way analysis of variance (ANOVA), with Tukey as post hoc, was used to check for significant differences among treatments. For non-normal data, the Kruskal–Wallis test was applied. Statistical differences were considered at *p* < 0.05. All analyses were performed using the SPSS 17.0 statistical package.

## Results

### Characterization of CS

Lipophilic pigments and antioxidant composition were analyzed in the CS of the three ferns species in their sporangial state. All contained the same six major carotenoids (neoxanthin, violaxanthin, antheraxanthin, lutein, zeaxanthin, and β-carotene) as typical for photosynthetic tissues, but their proportion differed among the three species. Carotenoids to Chl ratios showed a general pattern, being higher in *ETe* than in the other two species. The VAZ pigments, β-C concentrations, and the ratio AZ/VAZ was highest in *ETe*. The highest ratio Chla/b was found again in *ETe*, with significant differences with *ORe* and *MSt* (**Table 1**).

**Table 1:** Pigment composition and maximum photochemical efficiency of photosystem II (Fv/Fm) at the sporangial state in CS of the three studied ferns.

	*ORe*	*MSt*	*ETe*
**Chl a/b**	2.80 ± 0.07 a	2.71 ± 0.02 a	3.18 ± 0.01 b
**AZ/VAZ**	0.21 ± 0.01 a	0.16 ± 0.0 b	0.25 ± 0.01 c
**N**	52.5 ± 2.2 a	52.2 ± 1.4 a	49.9 ± 1.4 a
**V**	60.1 ± 6.4 a	81.8 ± 2.8 b	79.9 ± 2.8 b
**A**	5.26 ± 0.49 a	5.74 ± 0.29 a	14.3 ± 0.9 b
**L**	226.1 ± 13.3 a	226.1 ± 3.97 a	283.1 ± 6.1 b
**Z**	10.5 ± 0.5 a	9.6 ± 0.3 a	12.5 ± 1.0 b
**β-C**	96.7 ± 11.7ab	95.4 ± 3.2a	105.8 ± 2.2b
**VAZ**	75.9 ± 7.3 a	97.2 ± 3.1 b	106.8 ± 4.1 b
**Fv/Fm**	n.d	n.d.	0.131 ± 0.066

Results are expressed as follows: chlorophyll a/b ratio (Chl a/b) and AZ/VAZ ratio (mol mol−1). Neoxanthin (N), violaxanthin (V), antheraxanthin (A), lutein (L), zeaxanthin (Z), *β*-carotene (*β*-C), and VAZ (mmol mol−1 Chla + b). Each value represents the mean ± SE (n = 5 for pigments and n = 4 for Fv/Fm). Different letters indicate significant differences between species (P < 0.05). The term “n.d” means that the fluorescence signal is below the detection threshold.

To investigate the antioxidant capacity of the CS studied, the composition of the tocopherol pool and proline contents were examined in their sporangial state when they are released from sporangium. α-Tocopherol was ubiquitous among all the species. The lowest concentration of this antioxidant was found in *ORe*, while *MSt* had almost threefold higher values. Additionally, only the latter contained the other isoforms (β-, δ- and γ-) that represented 25% of the total tocopherol pool. 

The highest proline contents were also found in *MSt* CS. The amount of this amino acid showed great differences among the species. *MSt* CS accumulated proline in the highest proportion once mature, having twofold higher proline contents than *ORe* and sevenfold higher than *ETe* (**Table 2**). In summary, *MSt* presented the highest capacity of the antioxidant system.

**Table 2:** Tocopherol and proline contents at the sporangial state in the spores of the three studied ferns.

	*ORe*	*MSt*	*ETe*
**α-T**	0.30 ± 0.01 a	0.83 ± 0.01 b	0.43 ± 0.02 c
**(β + γ)-T**	n.d.	0.25 ± 0.00	n.d.
**δ-T**	n.d.	0.04 ± 0.00	n.d.
**Proline**	17.5 ± 2.7 a	35.7 ± 1.3 b	4.9 ± 0.1 c

Results are expressed as follows: α-tocopherol (α-T); β- + γ-tocopherol (β + γ-T); δ-tocopherol (δ-T) and proline (μmol g DW^−1^). Each value represents the mean ± SE (n = 5). Different letters indicate significant differences between species (P < 0.05). The term “n.d.” means that the concentration is below the detection threshold.

### Extent of Desiccation Tolerance

**Figure 1:**
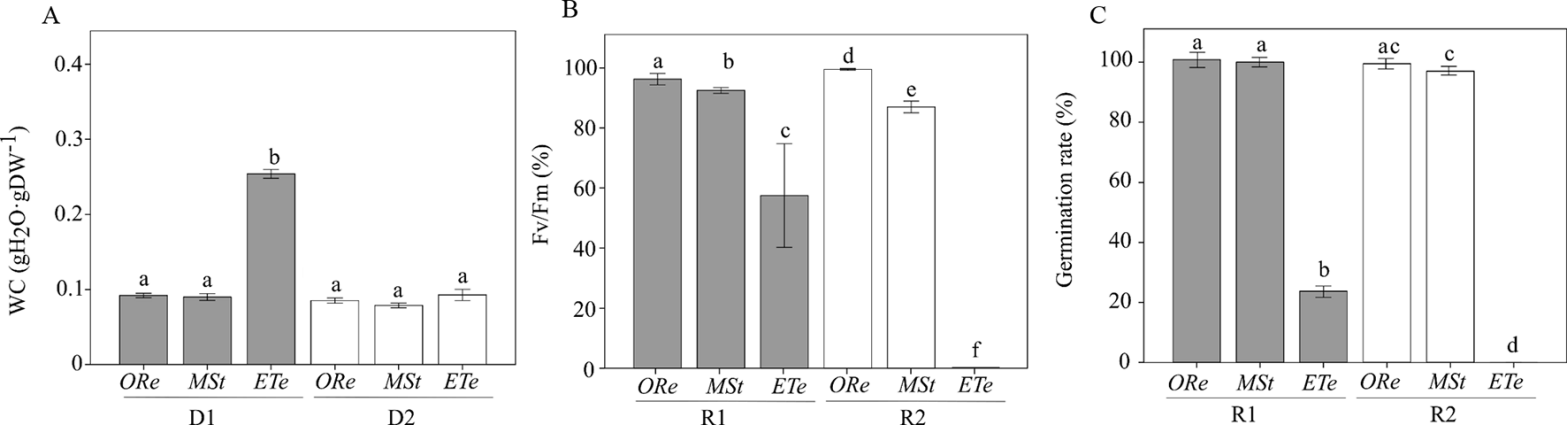
Water content (WC) **(A)**, percentage of recovery of Fv/Fm **(B)**, and percentage of germination **(C)** relative to the respective control values after first rehydration (R1) (gray bars) and second rehydration (R2) (white bars) at 10% RH in darkness in the spores of the three studied ferns. Control values of Fv/Fm in nontreated spores were obtained after 24 h of hydration in darkness. Control values of germination in nontreated spores were obtained 10 days after sowing on Dyer medium. Each bar represents the mean ± SE (*n* = 4). Different letters indicate significant differences among species and dehydration–rehydration (D–H) cycles (*P* < 0.05).

Mature CS of the three fern species were subjected twice to a D–R cycle at 10% RH in darkness. After D1, the WC greatly differed among species, with *ETe* able to retain the highest amount of water, while the other two species did not show significant differences (**Figure 1A**). After D2, no differences were observed among species, and WC values were below 0.1 g H_2_O g DW^−1^ for all the species studied. 

Regarding the capacity to tolerate desiccation (estimated by the recovery of Fv/Fm), *ORe* showed the highest values of Fv/Fm recovery after D1 and D2. Curiously, significant differences were found between both desiccations, being Fv/Fm recovery and germination percentage even higher after D2 than after D1 (**Figures 1B, C**). The next species that showed the highest recovery capacity were *MSt* CS. After the first drying, a recovery of Fv/Fm of 92% was possible, against the 87% recovery that occurred after D2. Finally, in the case of *ETe*, a reduction of almost 43% of Fv/Fm control values was observed after D1, and no recovery was possible after D2 (**Figure 1B**). In conclusion, all species were able to recover a certain percentage of Fv/Fm, at least after D1, but to a different extent. On the other hand, D2 treatment allowed the recovery of Fv/Fm only in *ORe* and *MSt*. Germination percentage, although with little differences with Fv/Fm recovery values, followed the same tendency. *ETe* showed a severe decrease in viability after D1 and no CS germinated after D2 (**Figure 1C**). 

**Figure 2:**
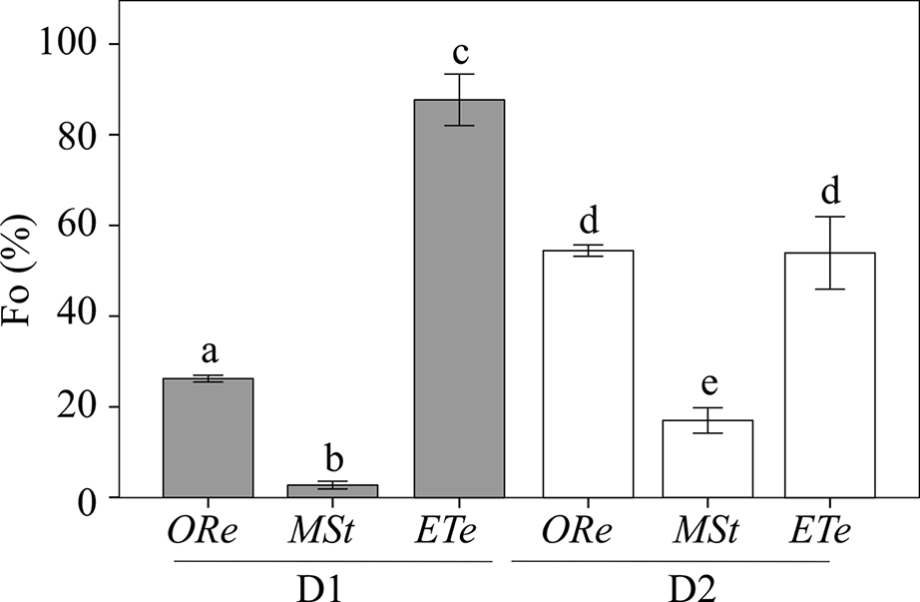
Percentage of Fo relative to the respective control values in the CS of the three studied ferns after desiccation (D1) (gray bars) and second desiccation (D2) (white bars). Control values were obtained as in **Figure 1**. Each bar represents the mean ± SE (*n* = 4). Different letters indicate significant differences among species and dehydration–rehydration (D–R) cycles (*P* < 0.05).

Concomitant with Fv/Fm changes, the basal fluorescence of chlorophyll (Fo) also underwent important changes during the D–R cycles, and completely different behaviors were observed among species. CS of *MSt* showed the highest quenching of chlorophyll upon desiccation with a reduction of 98% of the Fo in D1 and ∼83% in D2. On the contrary, *ETe* CS did not show such attenuation of chlorophyll fluorescence, neither in D1 or D2, although a greater quenching was observed during the D2. Finally, *ORe* increased the quenching of Fo much more during D1. After D2, Fo emission increased to values 50% lower than the controls (**Figure 2**).

### Molecular Mobility of CS in the Dry State

**Figure 3:**
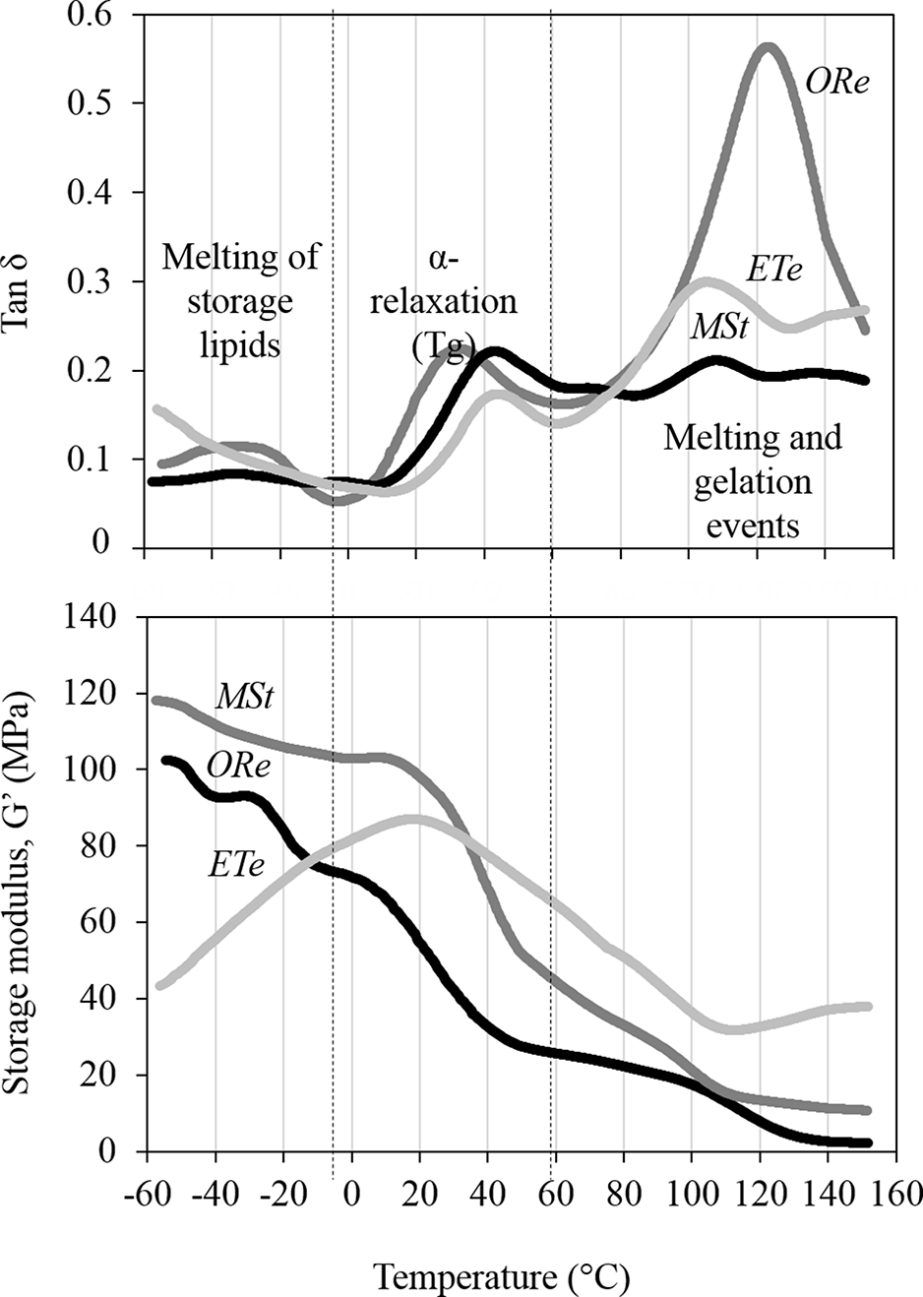
Dynamic mechanical thermal analysis (DMTA) scans of *ORe*, *MSt*, and *ETe* CS desiccated at 10% RH for 48 h. α-Relaxation is characterized by a peak in the tan δ between 30 and 50°C (upper panel) that coincides with a large decrease in storage modulus (G’) (lower panel). α-Relaxation coincides in temperature with the glass transition temperature (Tg) measured by differential scanning calorimetry (Ballesteros et al., 2017). Other molecular relaxations are observed above and below the Tg that are associated to melting and gelation events (as from Ballesteros and Walters, 2011) and melting of the storage lipids (Ballesteros et al., 2017), respectively. One representative curve (from *n* = 3 independent biological replicates) is shown for each spore species.

Molecular mobility in CS was estimated by DMTA. Spores were equilibrated for 48 h at 10% RH in darkness. Water contents achieved after this treatment were 0.055 ± 0.004, 0.051 ± 0.002, and 0.057 ± 0.00 g H_2_O g^−1^ DW for *ORe*, MSt, and *ETe* CS, respectively, with no significant differences between species. At these water contents, diverse structural relaxations were observed in the DMTA scans that were characterized by peaks in the tan δ and step-wise changes in the storage modulus (G’) (**Figure 3**). A relatively large relaxation was observed in the scans of all three species between 0 and 50°C (**Figure 3**). This relaxation was named as α-relaxation, and it was characterized by a relatively large peak in the tan δ (upper panel, **Figure 3**) that coincided with a large decrease in storage modulus (G’) (lower panel, **Figure 3**). α‐Relaxation coincides in temperature with the glass transition temperature (Tg) measured by differential scanning calorimetry in these three species at equivalent water contents (Ballesteros et al., 2017) and was considered the Tg of the CS in the three species studied. α-Relaxation or Tg occurred at 39.8 ± 2.3, 43.5 ± 1.8, and 42.2 ± 5.3°C for *ETe*, *MSt*, and *ORe* CS, respectively, with no significant differences between species (**Figure 3**). The size of the α‐relaxation or Tg in the tan δ (value related to the molecular mobility released during the relaxation, Ballesteros and Walters, 2011) was 0.08 ± 0.03, 0.17 ± 0.03, and 0.19 ± 0.09 for *ETe*, *MSt*, and *ORe* CS, respectively, being significantly different in the *ETe* CS when compared to those of *MSt* and *ORe*.

Below Tg (lower temperatures), all CS are in the glassy state, but some molecular mobility was detected in the tan δ as small peaks between −40 and −20°C coincided with a decreases in G’ (in *MSt* and *Ore* CS only). Interestingly, right before the α‐relaxation or Tg starts, the G’ of *ETe* increased dramatically (in all replicates performed), an event that did not occur in the other two species.

As the temperature increases above the Tg, the spore cytoplasm enters in a fluid state where molecular mobility increases greatly and chemical reactions are possible, including gelation and melting events of the starch and proteins that are characterized by large peaks of tan δ > 100°C, accompanied sometimes (e.g., *ETe*) with increases in G’ (detailed explanations of these events in Ballesteros and Walters, 2011).

### P–V Curves

**Figure 4:**
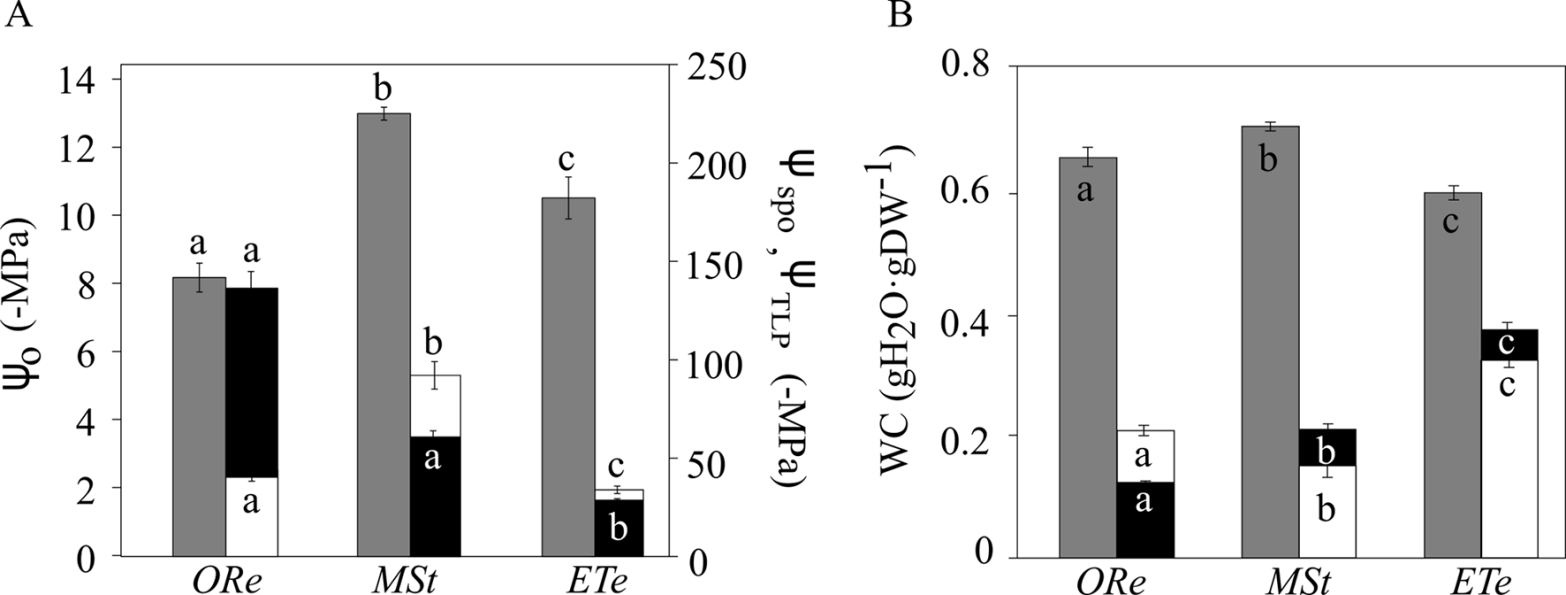
Water potential **(A)** and water content (WC) **(B)** in the spores of the three studied ferns at full turgor, at turgor loss point, and in the sporangial state. Gray bars correspond to water potentials at full turgor (Ψ_O_) (left axis), white bars correspond to water potential at turgor loss point (TLP) (Ψ_TLP_), and black bars correspond to water potential in the sporangial state (Ψ_SPO_) (right axis). The same color code is used for water content (WC). Each bar represents the mean ± SE (*n* = 3). Different letters indicate significant differences among species for the same parameter (*P* < 0.05).

Water potentials were measured during the loss of water in the CS of the three fern species equilibrated for 48 h at 100% RH. When spores were released from the sporangium, there were important differences with respect to their water potentials (Ψ_SPO_). *ORe* and *MSt* CS were released with a Ψ_SPO_ of −123 ± 10 MPa and −67 ± 3, respectively. In contrast, Ψ_SPO_ was much higher in *ETe* (−28 ± 4 MPa). Considering that the water potential at turgor loss point (Ψ_TLP_) was −44 ± 5, −92 ± 6, and −33 ± 2 MPa in *Ore*, *MSt*, and *ETe* CS, respectively, it can be concluded that spores of *ORe* are released from the sporangium in a “nonturgid” state (**Figure 4A**). As can be observed in **Figure 4A**, in the case of CS of *ETe*, the range between Ψ_SPO_ and Ψ_TLP_ is very narrow but enough to release the spore in a turgid state. When spores are at full turgor, the water potential did not reflect these differences.

Regarding the amount of water at full turgor (SWC), *MSt* showed the highest SWC, as well as the lowest WC at the turgor lost point (WC_TLP_). At the other extreme, *ETe* had the narrowest range of WC between the full turgor and the loss of turgor (**Figure 4B**). *ORe* spores, with an average of the modulus of elasticity (Ɛ) of 3.4 ± 0.9, showed the most flexible wall and was significantly different from the other two species. In the case of *MSt* and *ETe*, the wall was more rigid with Ɛ of 7.8 ± 0.6 and 6.3 ± 0.2, respectively, and even some cracks were observed in the coat of *MSt* (**Figure 6**).

### Water Uptake Capacity

The wettability of the spores was measured in their sporangial state with the aim of knowing their ability to absorb water once they are released from the sporangium.

**Figure 5:**
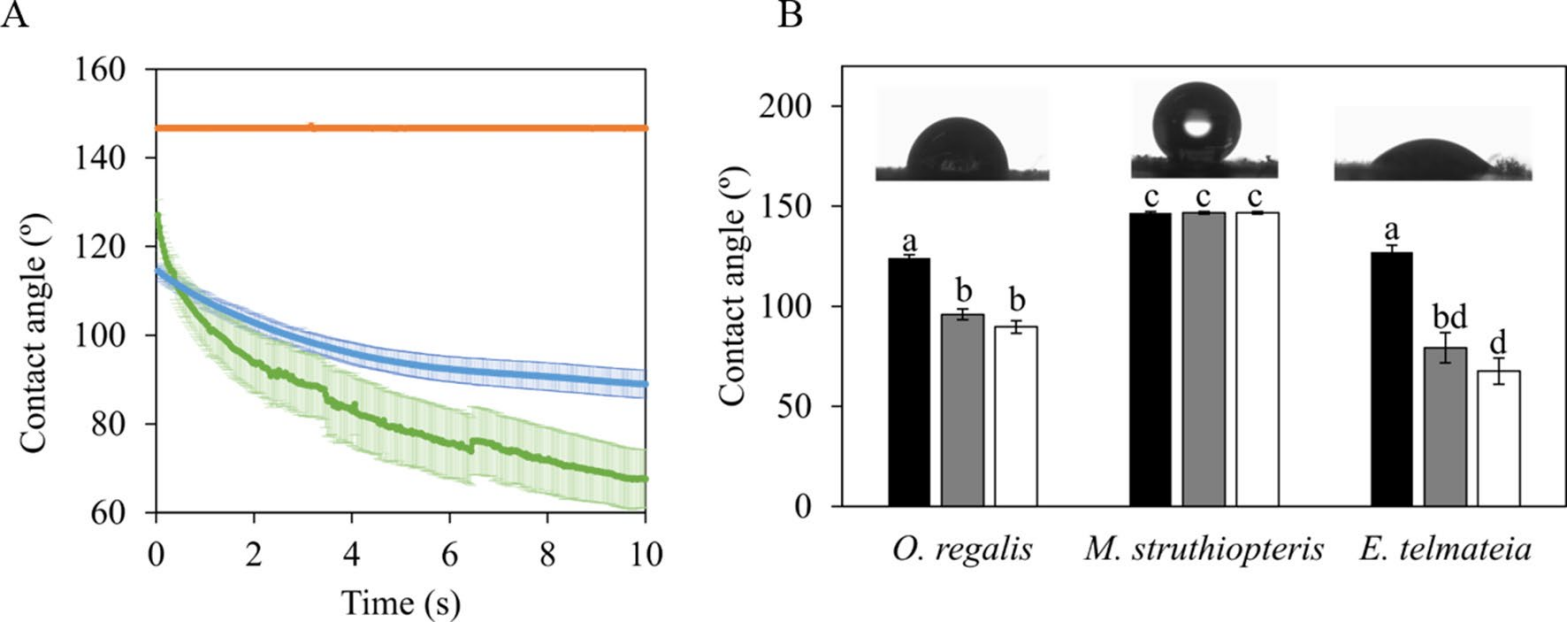
Static contact angles of 6 µl distilled water drops over the spores of the three studied species in their sporangial state.** (A)** Time course of the change in the contact angle during 10 s. Color lines represent the average ± SE (*n* = 10) for each of the three species evaluated *MSt* (orange line), *ORe* (blue line), and *ETe* (green line). Contact angles at selected time points during the recording: 1 s (black bars), 5 s (gray bars), and 10 s (white bars) after water drop contacted with the spore surface **(B)**. Each bar represents the mean ± SE (*n* = 10). Different letters indicate significant differences among species and time points (*P* < 0.05). Details of a representative distilled water drop in contact with spores surface at time 1 s are shown as insets.

Static CAs and how they changed with time are shown in **Figure 5**. The species that underwent the greatest changes in CA during 10 s (e.g., was more hydrophilic) was *ETe*. Drop CA changed from 127° ± 3° in the second 1, to 79° ± 7 in the second 5, and 60° ± 6 in the second 10. At the other extreme, no change was observed in *MSt* (**Figure 5A**). This species presented a hydrophobic behavior without significant differences in CAs between the three time points (**Figure 5B**). It should be noted that *ORe* had a lower rate of water absorption than *ETe*, despite starting with similar CA degrees.

### Outer Structure of the Spores

**Figure 6:**
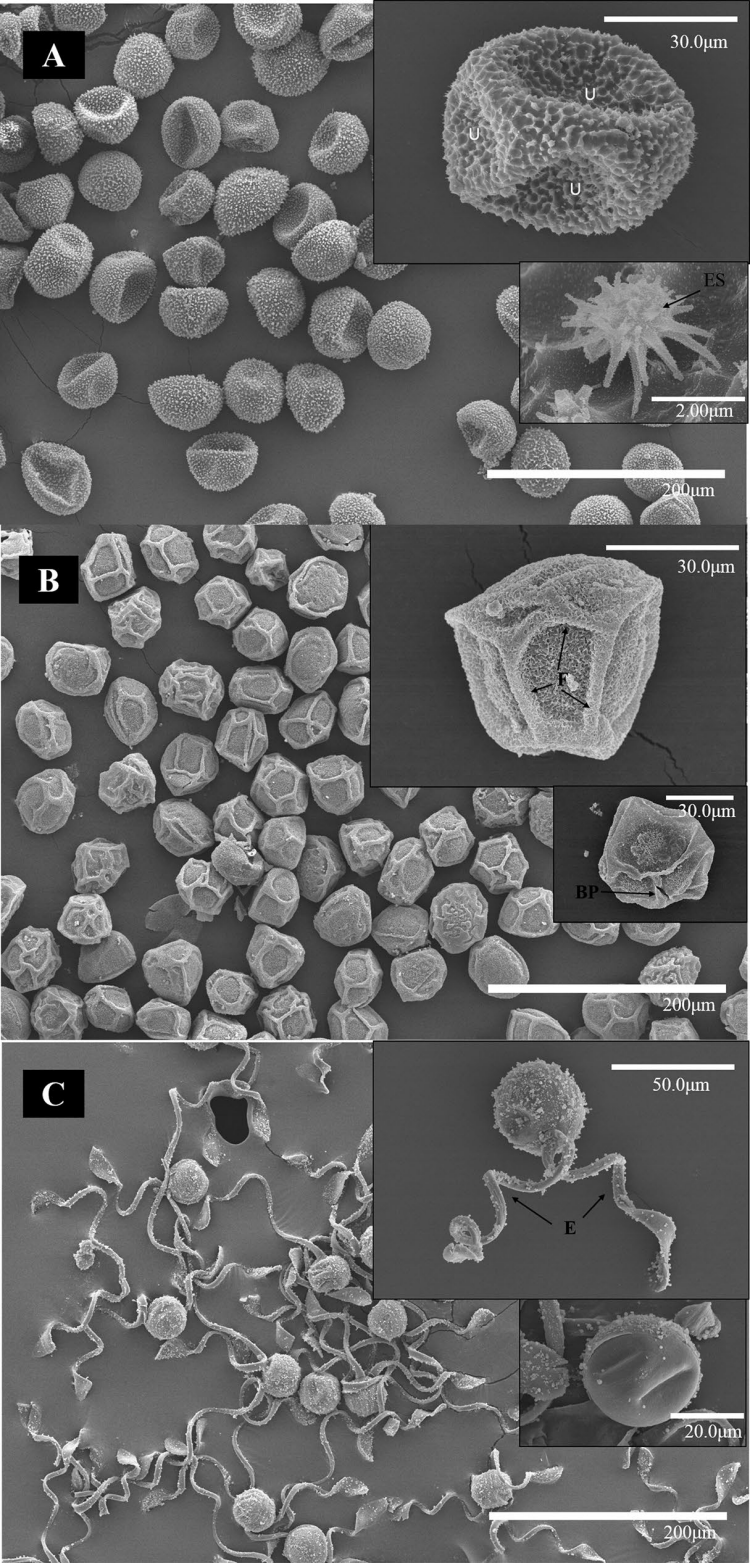
SEM micrographs of spores of *ORe* **(A)**, *MSt* **(B)**, and *ETe* **(C)** dried in silica gel during 24 h. The scale of each micrograph is indicated in the lower part. Abbreviations: (BP), broken perispore (E),elaters; (F), folds; U undulations.

The outer structure of the spores was studied by SEM. The *ORe* CS had a diameter of ∼40 μm and a rugose perispore with echinate structures (**Figure 6A**). These were homogeneously distributed along the entire spore surface. Several undulations were also apparent in the spore coat. *MSt* CS measured ∼30 μm, had coarse folds, prominent, and minutely echinate–rugose surface structures fused at the perispore. A break in the perispore can be observed in **Figure 6B**. *ETe* CS presented fewer amounts of peripheral structures associated with the perispore, but spherical structures could be observed (**Figure 6C**) both in spore surface and in elaters. Each spore measured ∼30 μm and had four paddle-shaped elaters. Only one pair is presented in **Figure 6C**.

## Discussion

In this study, we detected significant variation in the physiological and physicochemical properties of CS of three ferns species when subjected to D–R cycles, suggesting diverse extents of DT among CS. Based on the different ecological niches that these species occupy, this variation in DT may represent different adaptive strategies in terms of spore dispersal and subsequent sporophyte development. To date, no studies have investigated how CS respond to environmental conditions.

### Extent of DT Among CS

All mature CS of the three fern species studied showed a certain extent of DT since they were able to recover Fv/Fm and germinate once rehydrated. This feature, in more or less extent, has been commonly found in diverse CS (Ballesteros and Walters, 2011, Ballesteros et al., 2017; López-Pozo et al., 2019). However, despite the ability to recover metabolism after desiccation, several differences were observed between species that suggest different levels of DT. For example, the viability of *ETe* spores significantly decreased during D1, whereas *ORe* and *MSt* spores maintained their high initial viability (**Figure 1**). This initial reduction in viability occurred at higher water contents in *ETe* spores (∼0.25 g H_2_O g DW^−1^) than those reached in *ORe* and *MSt* spores (<0.10 g H_2_O g DW^−1^) (**Figure 1**). It is well known that the genus *Equisetum* presents short longevity in the dry state, whereas *ORe* and *MSt* can persist for months or years, respectively (Lloyd and Klekowski, 1970; Whittier, 1996; Lebkuecher, 1997; Ballesteros et al., 2017). However, the extremely fast viability loss in *ETe* and the high-water contents at which it occurred contrast with the relatively low water contents that other *Equisetum* species can tolerate for longer periods. In this sense, *Equisetum hyemale* can tolerate desiccation between 0.03 and 0.08 g H_2_O g DW^−1^ during 1 week without significant viability losses (Lebkuecher, 1997; Ballesteros et al., 2017). Our results showing large viability decrease in 36 h at 0.25 g H_2_O g DW^−1^ suggest a deficient DT of the spores of *ETe*, which resembles the DS found in recalcitrant seeds (Berjak and Pammenter, 2008; Walters, 2015).

Although all fern spores studied presented certain recovery of Fv/Fm (at least in R1), only spores of *MSt* and *ORe* carried out the characteristic NPQd of DT cryptogams. This phenomenon has been described widely in algae, cyanobacteria, lichens, and bryophytes (Bilger, 2014), but it has only been observed once in CS (López-Pozo et al., 2019). During desiccation, DT organisms that activate NPQd mechanism experience a strong decline in Fo. Instead, in DS organisms, an increment in Fo is noted. This process allows the dissipation of light energy in the dried state helping to limit oxidative risk. The poor (or the lack of) NPQd activation in *ETe* spores is another characteristic supporting the low DT or DS that they display (**Figure 2**).

### Extent of DT of CS Based on Pigments and Antioxidant System

The analysis of the pigment composition showed that all CS studied consist of the photosynthetic pigments characteristic of embryophytes (Esteban et al., 2015). *ETe* CS showed a higher Chl a/b ratio and were only photochemically active when released from sporangium (**Table 1**). At the other extreme, *MSt* had the lowest Chla/b and AZ/VAZ ratio, as well as β-C. Carotenoids are effective quenchers of ROS, thereby playing an important role in photosynthetic machinery protection (Demmig-Adams, 1990). The loss of viability of CS has always been related to the presence of chlorophyll, the absence of DT, the high metabolic rates, and the lack of dormancy (Kato, 1976). As CS possess fully developed photosynthetic apparatus and chlorophyll absorbs light even in the dry state, the risk of photodamage is exacerbated under these conditions. In this sense, CS have the behavior of any photosynthetic tissue and must cope with the high probability of photodamage (Ballesteros et al., 2018; López-Pozo et al., 2019). Not only pigments but also antioxidant system (tocopherols and proline) showed significant differences between species (**Table 2**). *ETe* and *ORe* contained only α-tocopherol, whereas *MSt* had additionally the other isoforms (β + γ)-tocopherol and δ-tocopherol. The photoprotective role of α-tocopherol is well documented (Munné-Bosch and Alegre, 2002) and is very common in photosynthetic tissues. The other isoforms β and δ are quite rare in most plant species, and γ-tocopherol is more common in seeds (Grusak and DellaPenna, 1999; Fernández-Marín et al. 2017). Abbasi et al. (2007) found that tobacco seeds increased the amounts of α-tocopherol as DT was acquired during maturation. Proline has also been described as a good ROS scavenger and compatible solute under stress conditions (Borsani et al., 1999). This amino acid could increase the antioxidant enzymatic capacity (e.g., APX, SOD, and CAT enzymes) under water stress conditions (de Campos et al., 2011). Proline would avoid or reduce lipids peroxidation and the production of free radicals. Besides, it would help in the scavenging of singlet oxygen and hydroxyl radicals (Kishor and Sreenivasulu, 2014). In this sense, *MTs* spores presented the highest concentration of this antioxidant, while *ETe* almost did not accumulate it (**Table 2**), supporting the DT difference between these species from an antioxidant point of view.

Overall, the results on pigments and antioxidant system indicate that while *MSt* and *ORe* photoprotection relies mostly on antioxidant systems, *ETe* shows a chloroplast composition more prone to imminent photochemical activation. In all likelihood, *MSt* and *ORe* displayed a trade-off between lower photosynthesis and higher DT. 

### Variation of the Stability of the Glassy State and Its Relation to the Extent of DT in CS

All spores equilibrated at 10% RH for 48 h were below the Tg and entered in a glassy state at ambient temperature (20°C) (**Figure 3**), where it is considered that there are no chemical reactions due to the low molecular mobility (Ballesteros and Walters, 2011; Fernández-Marín et al., 2013). However, some molecular mobility was observed below the Tg. For example, some broad peaks in the tan δ were observed between −40 and −20°C. These peaks can be attributed to the melting of the spore storage lipids, as previously described and measured by differential scanning calorimetry in these three species (Ballesteros et al., 2017). While the molecular mobility attributed to storage lipids may not be relevant to the variation of DT, there are other mechanical properties that may be important. For example, G’ of *ETe* increased dramatically right before the α‐relaxation (Tg) occurs, an event that was not observed in the other two species (**Figure 3**). Increments of G’ before the Tg have been related to inter- and intramolecular rearrangements that can lead to microstructural changes and are considered important in the stability of other dry systems (Herrera-Kao and Aguilar-Vega, 1999; Menard, 1999; Champion et al., 2000; Roudaut et al., 2004; Ballesteros and Walters, 2011). The presence of these inter- and intramolecular rearrangements in *ETe* could be interpreted as a consequence of the formation of an unstable glassy state upon drying and cooling, which could lead to large pores and a quick relaxation of the glassy state once formed (Walters et al., 2010). The significantly smaller size of the α‐relaxation in *ETe* when compared to *ORe* and *MSt* could also be indicative of a glassy state in *ETe* that has collapsed and compacted during the inter- and intramolecular rearrangements. In the case of *MSt*, it is interesting to consider its stable glassy state together with the release of the spores during wintertime. Freezing produces dehydration at the cellular level, so the development of DT mechanisms and the formation of stable intracellular glasses are necessary for their long-term survival. Overall, these results are in agreement with a lower DT and shorter lifespan of *ETe* spores at low water content.

### The Loss of Turgor as a Parameter of Discrimination Between Species and Habitats

To understand how organisms respond to changes in water availability, the study of P–V curves offers a series of physiological parameters about water relations (Tyree and Hammel, 1972; Kikuta et al., 1985). These parameters have been widely studied in vascular plants, but little is known about poikilohydric organisms (Holzinger and Karsten, 2013, Nardini et al., 2013; Petruzzellis et al., 2018). Several parameters inferred from P–V curves of *MSt*, *ORe*, and *ETe* showed important differences that could be related to their ecological requirements (water availability) and physiological features (DT) (**Figure 4**). One of the greatest risks to the cell during water loss is the reduction in its volume (Walters, 2015). When the protoplast begins to dehydrate, this reduction in volume can threaten the integrity of the membrane–wall junctions. Therefore, the correct folding of the wall during desiccation is of vital importance to overcome the mechanical stress that occurs, avoiding the disruption of these connections (Fernández-Marín et al., 2016). The parameters that best describe the ability of the cells to maintain turgor are Ψ_O_, Ψ_TLP_, and Ɛ (Lenz et al., 2006; Ding et al., 2014). Of plants that tolerate drought, some of the most characteristic features within water relations are that they have lower Ψ_O_, which in turn is related to lower Ψ_TLP_ (Bartlett et al., 2012). *ETe*, the least DT, showed the highest Ψ_TLP_, whereas *ORe*, the most DT, showed the lowest Ψ_TLP_ (**Figure 4**). Not only was drought tolerance correlated with low Ψ_TLP_, but environmental water availability in the sporulation moment also correlated with this parameter. *ORe*, followed by *MSt*, had the lowest Ψ_TLP_, coinciding with times of the year with lower water availability. Spores of *ETe* had the highest value of Ψ_TLP_, coinciding with the fact that this species releases its spores in the spring (**Figure 4**). The relationship between Ψ_TLP_ and environmental conditions has been demonstrated in vascular plants, where those that grow in climates with water deficit (those adapted to live in xeric climates) present lower Ψ_TLP_ values (Aranda et al., 1996, Lenz et al., 2006, Bartlett et al., 2012, Nardini et al., 2013). 

Regarding Ɛ, there is still no consensus to clarify whether low values are related to xeric climates or not (Abrams and Kubiske, 1994, Anderson and Helms, 1994, Saito and Terashima, 2004). It has been suggested that low values of Ɛ can help to maintain turgidity even when large amounts of water are lost (Schulte 1992); on the other hand, high values could help a rapid recovery of the turgidity. In organisms subjected to daily hydration–dehydration cycles, this could be a great advantage (Bidussi et al., 2013; Alam et al., 2015). Therefore, *ORe* could maintain the turgor through the high elasticity of the walls, whereas *MSt* may have a greater osmotic adjustment capacity. The maintenance of turgor could be based on compatible solutes (e.g., proline, **Table 2**) instead of Ɛ. By observing SEM images, the capacity of *ORe* to fold their wall can be appreciated (**Figure 6**). These undulations were not found in the *MSt* spores.

### Wettability. Dispersal Function or Unfavorable Conditions Avoidance?

How CS surfaces absorb water will determine to a great extent the physiological responses when released to the environment. Haines et al. (1985) found that environmental factors would more greatly affect the plant surface if the wettability was high, so the species would be more exposed to environmental factors. In agreement with this, the CS of *ETe* would be more affected by environmental conditions due to their higher wettability (**Figure 5**). On the other hand, *ORe* and *MSt* developed CS that were remarkably hydrophobic and thus with significantly lower wettability (**Figure 5**). Low wettability in elm samaras has been related to high floatability, which would help to a dispersal mechanism based in hydrochory (Guzmán-Delgado et al., 2017). Although a similar advantage for hydrochory could be argued in the case of *MSt*, the low permeability of its CS may also relate to a delay of spore germination in the field. As hydrating during winter could induce germination under unfavorable conditions, a delay in the absorption of water, activation of metabolism, and finally, germination may increase survival probabilities of developing gametophytes during late winter/early spring. *ORe* and *ETe* are released in more favorable atmospheric conditions, so their relatively faster capacity to take up water may be advantageous according to their phenologies. 

### Concluding Remarks

**Figure 7:**
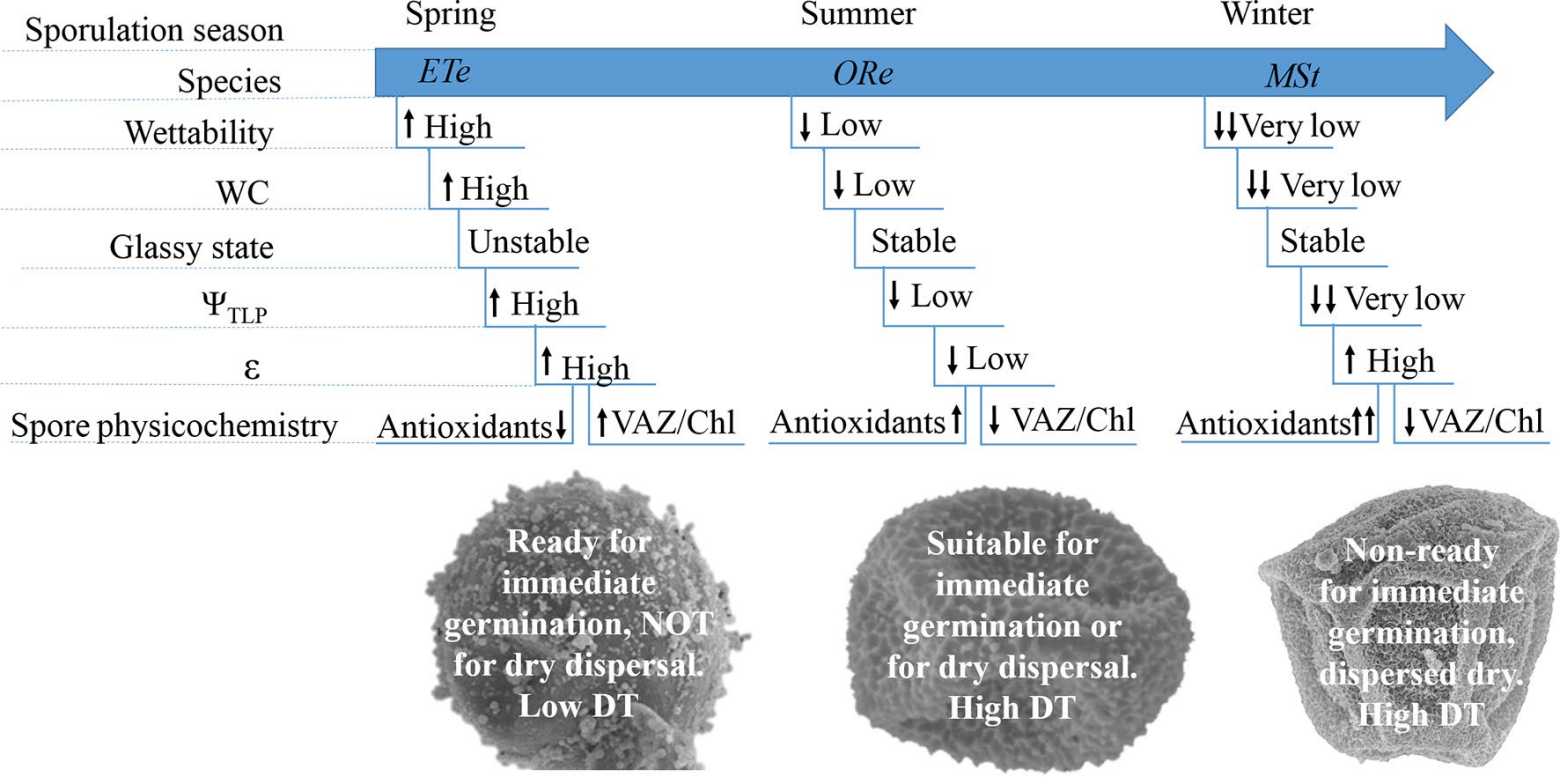
Summary view of the main trends on the physiological and physicochemical features in CS of the three ferns studied. Arrows indicate higher or lower values when each parameter is compared among species.

In view of the results, several conclusions can be achieved. CS of *ETe* had the highest capacity to absorb water, the lowest DT, and presented the most unstable glassy state when dried. Its pigment composition revealed effective photosynthetic machinery and WC, and Fv/Fm were higher than in the two other species. Since these spores are released in spring, when water availability is high and spores will germinate immediately after dispersal, this species does not require the development of DT strategies to survive for long periods in the dry state (**Figure 7**). These spores displayed a trade-off between photosynthesis and DT. In summer, when water availability fluctuations are more frequent, *ORe* releases its spores. Because of this, *ORe* is more likely to suffer various cycles of D–R. Its spores had the highest DT and wall flexibility and the lowest Ψ_TLP_ and Ψ_SPO_. All of these features enable *ORe* to dehydrate and rehydrate in their natural habitat once released without compromising viability (**Figure 7**). Finally, *MSt* had the highest hydrophobicity, highest antioxidant system, and high DT. The release of this spore occurs in winter, under very unfavorable environmental conditions. The high hydrophobicity could prevent the entry of water under these conditions (**Figure 7**).

Overall, the coordination among several hydric, mechanical, physical, and physiological traits represents a syndrome that confer CS the level of desiccation tolerance required to survive under the conditions prevailing during the natural dispersion of each species.

## Data Availability

The datasets generated for this study are available on request to the corresponding author.

## Author Contributions

ML-P designed the experimental protocol, conducted the experimental phase and statistical analyses, and drafted the manuscript with support from BF-M and JG-P. DB contributed to the interpretation of DMA data and in the discussion of overall results. JL performed the DMA analyses with contributions of BF-M and ML-P. JG-P designed the experimental protocol and supervised the writing of the manuscript. BF-M designed the experimental protocol and supervised the writing of the manuscript. All coauthors contributed to the final version of the work.

## Funding

This work was funded by (i) the Basque Government (research project UPV/EHU IT-1018-16, UPV/EHU IT-718-13, and Predoctoral Fellowship to MLP); (ii) Royal Botanic Gardens Kew receives grant-in-aid from Defra. Spanish Ministry of Science, Innovation and Universities (MCIU/FEDER, UE) (PGC2018-093824-B-C44).

Eusko Jaurlaritza (Award number(s): UPV/EHU IT-1018-16); Eusko Jaurlaritza (Award number(s): UPV/EHU IT-718-13); Department for Environment, Food and Rural Affairs (Award number(s): Royal Botanic Gardens Kew receives grant-in-aid from Defra); Eusko Jaurlaritza (Award number(s): Predoctoral Fellowship to MLP); Spanish Ministry of Science, Innovation and Universities (MCIU / FEDER, EU) (PGC2018-093824-B-C44).

## Conflict of Interest Statement

The authors declare that the research was conducted in the absence of any commercial or financial relationships that could be construed as a potential conflict of interest.

## Abbreviations

AZ/VAZ, de-epoxidation state of the xanthophyll cycle; β-C, β-carotene; Chl, chlorophyll; CS, chlorophyllous spores; D1, first dehydration; D2, second dehydration; D, desiccation; DMTA, dynamic mechanical thermal analyzer; D–R, dehydration–rehydration; DS, desiccation sensitive; DT, desiccation tolerance; DW, dry weight; ε, elasticity modulus; *ETe*, *Equisetum telmateia*; EW, equilibrium weight; Fm, maximum chlorophyll fluorescence; Fo, minimum chlorophyll fluorescence; Fv, variable chlorophyll fluorescence; Fv/Fm, maximum photochemical efficiency of photosystem II; G´, storage modulus; *MSt*, *Matteuccia struthiopteris*; NPQd, desiccation-induced quenching of chlorophyll fluorescence; *ORe*, *Osmunda regalis*; PSII, photosystem II; P–V, pressure–volume; R1, first rehydration; R2, second rehydration; R, rehydration; RH, relative humidity; ROS, reactive oxygen species; RWC_tlp_, relative water content at turgor lost point; SWC, saturated water content; tan δ, loss tangent; Tg, glass transition temperature; VAZ, violaxanthin + anteraxanthin + zeaxanthin; Ψ, water potential; Ψ_o_, osmotic water potential; Ψ_spo_, sporangial water potential; Ψ_tlp_ water potential at turgor lost point
